# Influence of reinforcement learning on the inhibitory control of Internet gaming disorder

**DOI:** 10.1002/pchj.772

**Published:** 2024-07-05

**Authors:** Mengyue Zhang, Chenyue Zhao, Meng Zhang, Shuangshuang Mao, Mengyao Yang, Ziyu Mao, Xiaoli Xing

**Affiliations:** ^1^ Department of Psychology Henan University Kaifeng China; ^2^ Department of Basic Education Hunan Vocational College for Nationalities Yueyang China

**Keywords:** inhibitory control, Internet gaming disorder, reinforcement learning

## Abstract

Reward processing dysfunction and inhibition control deficiency have been observed in Internet gaming disorder (IGD). However, it is still unclear whether the previous reinforcement learning depends on reward/punishment feedback influences on the cognitive inhibitory control of IGD. This study compared the differences between an IGD group and healthy people without game experiences in the probability selection task and the subsequent stop signal task by the method of behavioral experiments, in order to explore whether the reward learning ability is impaired in the IGD group. We also discuss the influence of previous reward learning on subsequent inhibition control. The results showed that (1) during the reward learning phase, the IGD group's accuracy was significantly lower than that of the control group; (2) compared with the control group, the IGD group's reaction times were longer in the transfer phase; (3) for no‐go trials of the inhibitory control phase after reward learning, the accuracy of the reward‐related stimulation in the IGD group was lower than that of punishment‐related or neutral stimulation, but there was no significant difference among the three conditions in the control group. These findings indicated that the reinforcement learning ability of the IGD group was impaired, which further caused the abnormal response to reinforcement stimuli.

## INTRODUCTION

Internet gaming disorder (IGD) means that individuals seek physical and psychological pleasure in online games, and cannot control their own behavior so much as to result in a pathological online use behavior. Individuals with IGD show symptoms of concentration, tolerance and withdrawal and have been addicted to online games for a long time. The condition causes a series of negative effects on study and life, including lower subjective well‐being and more negative emotions (Ostovar et al., 2016), which are highly comorbid with depression and anxiety (Gentile, 2009; Van Rooij et al., 2014). Healthy game players are individuals who have game experiences but are not addicted to online games, and the gaming behaviors have no negative impact on their physical and mental well‐being. IGD was included in the Diagnostic and Statistical Manual of Mental Disorders (Fifth Edition, Text Revision; DSM‐5‐TR; American Psychological Association, 2022) as a condition for further study. Similar to drug addiction, gambling disorder, and other addiction disorders, there are also some symptoms of concentration, tolerance and withdrawal in IGD (Petry et al., 2014). At the same time, IGD and other types of addiction disorder all cause cognitive dysfunction, including abnormal reward function, impaired decision‐making function and inhibitory control behaviors (Antons et al., 2020; Balconi et al., 2014; Cabedo‐Peris et al., 2022; García‐García et al., 2014; Luke & Goudriaan, 2018; Wang et al., 2023; Zheng et al., 2022). These cognitive dysfunctions are manifested in several brain regions and system activation abnormalities shown in both substance addiction and behavioral addiction, including the prefrontal cortex, amygdala, hippocampus and mesolimbic dopamine system (Lu et al., 2005; Mantsch et al., 2016; Siciliano et al., 2019).

Reinforcement learning is an ability to seek rewards and avoid losses in an uncertain environment, and external feedback is a necessary information prompt to adjust behavior in reinforcement learning (Muller‐Gass et al., 2017), which is essential for adaptive functioning. Impaired daily reinforcement learning is an important aspect of addiction pathology, and also has practical and theoretical meaningfulness (Volkow et al., 2016). Many investigations found that abusers of substances (such as alcohol, marijuana, and nicotine) perform poorly in reinforcing learning from rewards and losses (Baker et al., 2011, 2013; Rai et al., 2019). The reason may be that their long‐term “drug” exposure may desensitize dopamine system and reduce reward sensitivity. The research of Baker et al. (2013) supported this addiction model, and suggested that time would lead to desensitization of reward circuit.

It has been observed that the reward and loss sensitivity decreases in individuals with IGD (Li et al., 2020; Wang et al., 2020). Dopamine (DA), as the neurotransmitter, is essential for reward processing, and the decline of availability of dopamine receptor and dopamine transporter might prevent IGD patients from using clues to predict reward stimuli (Hou et al., 2012; Tian et al., 2014). Brain‐imaging studies show that reward processing involves the striatum, medial orbitofrontal cortex and anterior cingulate cortex (Yao et al., 2017). However, these brain regions are less activated by reward stimuli in individuals with IGD. For example, compared with the healthy control group with game experiences, reward cues were expected to cause less activation of the striatum in the IGD group (Hahn et al., 2014; Kim et al., 2014), and the activation degree of the right caudate nucleus, left orbitofrontal cortex, and dorsolateral prefrontal cortex was less for accidental reward in IGD participants (Lei et al., 2022). Meanwhile, electrophysiology research has obtained similar results. The amplitudes of feedback‐related negativity (FRN) to reward stimuli were observed to be lower in adolescents with IGD, indicating the weaker sensitivity of IGD participants to reward (Li et al., 2020). For loss cues, previous studies have shown that IGD participants were insensitive to loss (Wang et al., 2020); the amplitudes of FRN and P300 to loss stimuli were observed to be lower in IGD participants (Yau et al., 2015); and the activation of the anterior cingulate cortex in IGD participants decreased in response to negative feedback (Dong et al., 2011). These studies suggested that reward learning performance might be impaired in the IGD group. The reason might be that Internet addicts were insensitive to reward/punishment stimuli, which caused their failure to have an appropriate response to the outcome feedback of online games, therefore aggravating the use of them.

The previous reward learning could influence the subsequent cognitive processing of reward‐related stimuli in the new environment. The learning of stimulus–response‐outcome associations does not only lead to behavioral changes but also neural changes in the visual cortex. The “history of reward” guides visual attention in a rather automatic fashion (Theeuwes, 2018), which is advantageous as long as it is in line with our current goals and intentions. However, we also preferentially attend reward‐associated stimuli under conditions where it is no longer helpful or even entails negative consequences (Camara et al., 2013; Le Pelley et al., 2015). Stimuli with a history of reward can impact cognitive control, which enables planning and execution of advantageous behavior as well as the ability to manage conflicting information and inhibit undesirable responses in accordance with internal goals and intentions (Bühringer et al., 2008). However, individuals' decisions are slowed and less optimal when a task‐irrelevant but reward‐associated distractor is present (Gluth et al., 2018; Itthipuripat et al., 2015). For example, in a lateral inhibition task, when flankers not only conflict with the signal to go for a response, but are also associated with value, they interfere with otherwise typically found inhibitory processes altering response speed (Anderson et al., 2016; Kim & Anderson, 2019). Interference effects by task‐irrelevant but reward signal stimuli were also observed in a Stroop Task (Liao et al., 2020).

Both reward processing disorder and impaired executive function play important roles in addictive behavior. The dysfunction of brain regions on inhibiting reward seeking might be an important factor of IGD (Zhang et al., 2021). One recent study on IGD found that Internet addicts persisted in responding to the previous reward‐associative stimuli even if the feedback of reward did not appear in the habit test of the tool‐learning paradigm (Zhou et al., 2018), which indicated that the value of previous reward‐associated stimuli continued to influence the subsequent behavioral response of addicts. In spite of such above research, there is little knowledge of how the previous reward learning of IGD influences the subsequent inhibition control system.

This study aims to explore the reward learning ability of individuals with IGD and its influence on subsequent inhibition control. First, a probability‐selection task was used to quantify individual learning differences (Frank et al., 2004), and then the task of selecting a stop signal was employed to investigate the influences of previous reinforcement learning on inhibition control of IGD. Based on the above review, we hypothesized that individuals with IGD might perform worse in the reinforcement learning and subsequent inhibitory control task compared to a healthy control (HC) group.

## METHODS

### Participants

All participants were selected based on a modified Internet Addiction Test (IAT; Young, 1996) and the nine‐item diagnostic criteria in the *DSM‐5‐TR* (American Psychological Association, 2022). IGD participants scored higher than 50 on the modified IAT and concurrently met five or more *DSM‐5‐TR* criteria, and played Internet games for minimum 14 h per week during the last 2 years. The HC group scored lower than 50 on the modified IAT and met fewer than five *DSM‐5‐TR* criteria, and played online games for less than 1 h per day. All participants met the following criteria: right‐handed; normal or corrected‐to‐normal vision; and (prior to scanning) medication‐free and not using any substances (e.g., alcohol, nicotine/tobacco, and coffee).

According to the above criteria, we recruited 21 IGD participants (15 males, 6 females) and 24 HC participants (15 males, 9 females) through posters and online advertisements. Only the participants who completed the learning stage of the probabilistic selection task with AB pair accuracy exceeded 60% and CD accuracy exceeded 50% during the training period were included in the final analysis (Rai et al., 2019). Therefore, one participant was excluded from the IGD group and one was excluded from the HC group, and finally 20 participants from the IGD group and 23 participants from the HC group were included in the experimental analysis. Demographic information of the remaining participants is shown in Table 1. Age, education, and other measures were matched between the two groups.

**TABLE 1:** Data analysis results of self‐report questionnaire (*M* ± *SD*)

Variable	IGD (*n* = 21)	HC (*n* = 24)	*p*
Age	19.35 ± 0.98	19.77 ± 1.87	.373
*DSM‐5*	6.10 ± 1.04	0.83 ± 1.02	<.001
IAT	57.38 ± 9.14	28.00 ± 6.79	<.001
Game duration	22.04 ± 11.48	1.43 ± 1.52	<.001
Game craving	3.52 ± 3.01	0.82 ± 0.83	<.001

Abbreviations: DSM‐5, *Diagnostic and Statistical Manual of Mental Disorders*, 5th ed.; Game craving, self‐reported craving; HC, healthy control group; IAT, Internet Addiction Test; IGD, Internet gaming disorder group.

### Measures

The game usage questionnaire includes some basic surveys of the participants' game behaviors, including basic personal information, the weekly game duration in the last 12 months, the percentage of game behaviors in Internet usage, the game history, the three most played games in the last year, the average weekly use duration of each game and the use of tobacco and alcohol.

The *Diagnostic and Statistical Manual of Mental Disorders‐5 (DSM‐5*): each item corresponds to a symptom of online game addiction, and a score of more than 5 meets the *DSM‐5‐TR* online game addiction screening standard (Petry et al., 2014). Specially, the nine diagnostic criteria of *DSM‐5‐TR* are the same as the nine diagnostic criteria of *DSM‐5*.

The Internet Addiction Test (IAT) uses the adapted version of the Internet Addiction Questionnaire compiled by Young (1996) and Wang et al. (2017). The IAT is a self‐report instrument using a five‐point Likert scale (1=“hardly ever”; 2=“occasionally”; 3=“sometimes”; 4=“often”; 5=“always”) and contains 20 items, referring to previous studies (Dong et al., 2018; Wang et al., 2017). A score of more than 50 points means that there is a tendency to become addicted to online games. The study has proved that the modified Chinese version of the IAT is compatible with the nine diagnostic criteria of IGD in the *DSM‐5‐TR* and that the overall psychometric property of this Chinese version IAT is good (Lu et al., 2022).

The Game Craving Scale is developed by Tiffany and Christen (Cox et al., 2001), which consists of 10 items and is the Dichtomous scale. Participants evaluate whether it meets the situation described by each item according to the current state. The higher the score, the higher the craving for the game.

### Experimental procedure

**FIGURE 1:**
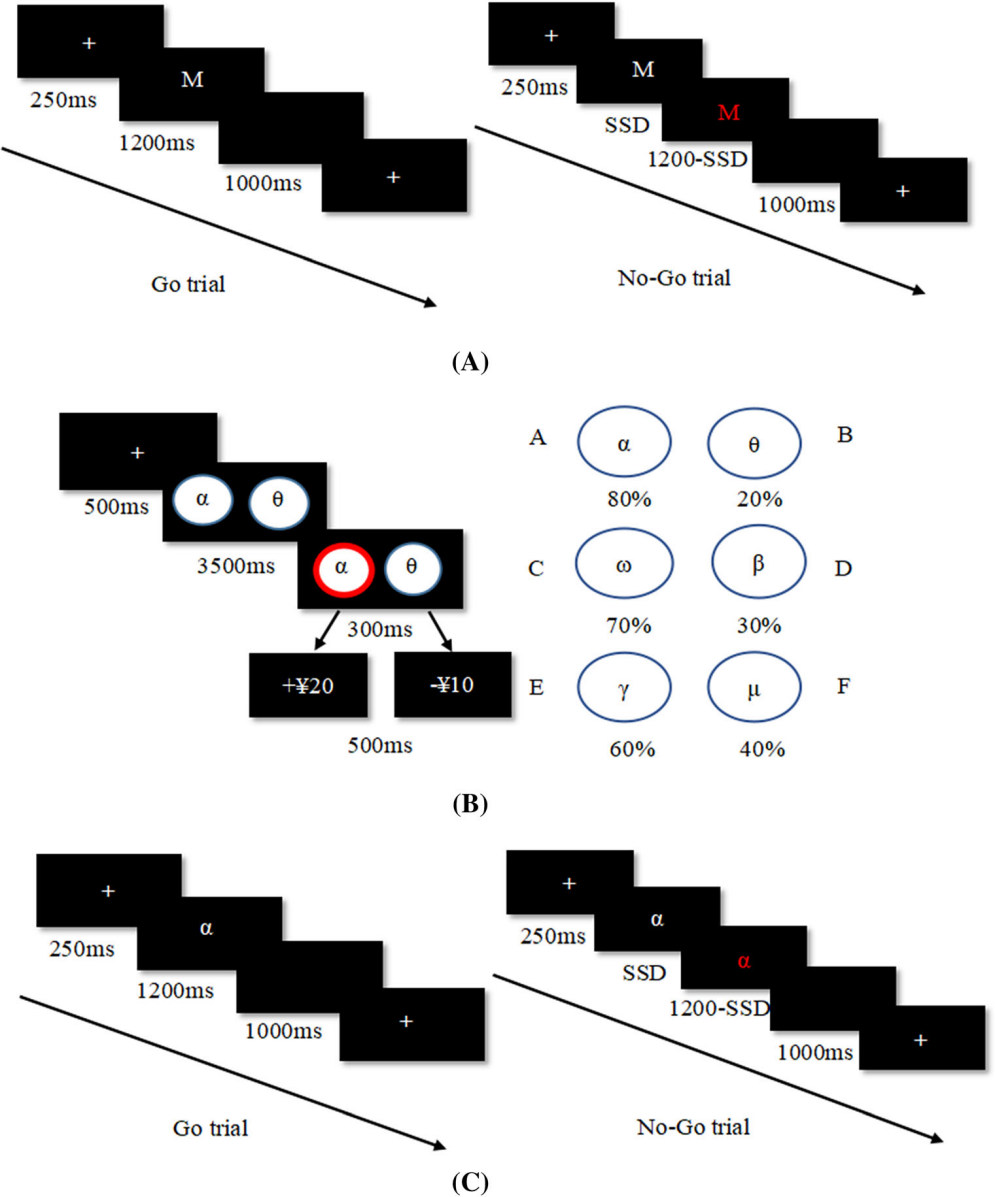
Flow chart of the experiment. (A) A single process of Go trial and a single process of No‐Go trial in neutral signal stop task. (B) A single flow chart of reward learning stage and three stimulus pairs. (C) A single process of Go trial and a single process of No‐Go trial in stop signal task of reward and punishment related stimulation

Programming was done with E‐prime2.0. Before the formal experiment, all the participants signed the informed consent form, and completed the Game Craving Scale. The neutral cue stop signal task, the probability selection task, and the reward and punishment stimulation stop signal task were completed after the connection was established in a quiet environment. The specific process is shown in Figure 1.

#### Stop signal task

In the stop signal task, the participant is required to press the key at the Go signal as quickly and accurately as possible, but inhibit the reaction when there is a Stop signal appears after the Go signal. The Stop signal appears only in a few Stop trials, and the time interval (stop signal delay [SSD]) between the Go signal and the Stop signal is set according to the tracking algorithm (Williams et al.,^1999^). In this study, the SSD was initially set to 300 ms, and then adjusted according to the performance of the participants: if the reaction was successfully suppressed in the current stop attempt, the SSD in the next stop attempt was increased by 50 ms to shift up the inhibition difficulty. If the participant failed to suppress, the SSD was reduced by 50 ms to decrease the suppression difficulty in the next trial. There were 24 practice trials before the formal experiment. Before the practice trials, the main test explained the experimental instructions. After the practice, the participants reported the task rules orally. If they failed to master them, they continued to practice until they managed to master them.

#### Neutral signal stop task

In the trials without reward and punishment, the Go trial started with a 250‐ms fixation point “+”; Then a white letter “M” or “N” (Go signal) appeared on a black background. If the letter “M” appeared in the center of the screen, the participant started pressing the “F” key. And if the letter “N” appeared in the center of the screen, the participant started pressing the “J” key. In Go trials, when the white character appeared, the participant needed to press the key during 1200 ms. In No‐Go trials, the white character lasted for SSD and then turned to red (Stop signal), and the participant needed to inhibit the key pressing. The red character (Stop signal) lasted for 1200‐SSD ms. When the character disappeared, there was an blank screen of 1000 ms, and then the next trial started. The experiment formally included 320 trials, including 240 trials of Go signal (120 trials of M and N each); And there are 80 No‐Go trials (40 trials of M and N each). Participants could take a rest after every 160 trials, and the rest time was determined by the participants (Figure 1A).

#### Probabilistic selection task

The probabilistic selection task was adapted by Frank et al. (2004). In each trial, a pair of characters appeared on the computer screen and the participant were asked to make a choice between the two characters to get reward (the correct choice) or to lose (the incorrect choice). In the reward learning phase, six Greek characters, hereinafter referred to as A–F (specifically, α was referred as stimulus A, θ as stimulus B, ω as stimulus C, β as stimulus D, γ as stimulus E, and μ as stimulus F), were divided into three pairs (AB, CD, and EF). The presented order of the stimulus pairs was counterbalanced. Participants were asked to choose a stimulus to reward 20 yuan (correct) or lose 10 yuan (incorrect) by pressing the left or right key on the computer keyboard. The reward probability of different stimulus pairs was different. In AB trials, a choice of A brought the financial victory in 80% of trials, while in 20% of the choices, choosing A would led to the financial loss. When B was selected, the winning/losing chance was opposite. It was more difficult to make accurate responses in CD trials and EF trials: the relative chances of winning and losing were 70/30 of choosing C in CD trials and 60/40 of choosing E in EF trials. If D was chosen in CD trials, the winning/losing chance will be contrary for C. And compared with E, the winning/losing rate of F selected was also opposite in EF trials. There were three blocks in the learning stage and each block included 40 trials per stimulus pair, resulting in a total of 120 trials in each block and a total of 360 trials in the learning stages (Figure 1B).

In the reward learning test phase, the same three stimulus pairs (AB, CD, and EF) were presented and participants need to make the choice between the learned pairs. But there was no feedback after the choice in the reward test stage. The test phase included 60 trials, 20 for each stimulus pair.

In the reward learning transfer phase, the six stimuli randomly formed new stimulus pairs. The participant were asked to make choices in the new stimulus pairs. Specially, compared to other stimuli (C‐F), A and B were respectively the “best” and “worst” stimuli in terms of reward probability (i.e., the trials had A or B respectively). The relative frequency of choosing stimulus A and avoiding stimulus B indicated that the participants had learned from positive feedback or negative feedback (Frank et al., 2004). The trials of the transfer phase did not involve feedback. The transfer phase included 160 trials, 20 for each new stimulus pair.

#### Stop signal task of reward and punishment related stimulation

The procedure in stop signal task of reward and punishment related stimulation is the same as the neutral stop signal task, only the high reward stimulation (α) and high loss stimulation (θ) learned in the conditioning phase (the probabilistic selection task) were showed in the inhibition control phase (Davidow et al., 2019). In each trial, a 250‐ms fixation point “+” was showed on the screen at the beginning. Then, a white character “α” or “θ” appeared on a black background in Go trials and the maximum duration is 1200 ms. When the character “α” appeared in the center of the screen, the participant started pressing the “F” key immediately. If the character “θ” appeared in the center of the screen, the participant started pressing the “J” key. In the No‐Go trials, the white character lasted for SSD and then became red character (Stop signal), and the pressing should be inhibited. The red character (Stop signal) lasted for 1200‐SSD ms. After the character disappeared, there was a blank screen of 1000 ms, and then the next trial started. The experiment phase officially included 320 trials, 240 trials of Go signal, including 120 reward‐stimulation trials and 120 punishment‐stimulation trials. The Stop signal was shown 80 times, and the reward stimulation and punishment stimulation for 40 times each in No‐Go trials. Participants could take a rest after every 160 trials, and the rest time was determined by the participants (Figure 1C).

### Statistical methods

SPSS 20.0 was used for statistical analysis (IBM SPSS Statistics). Independent sample*T*‐test was carried out on self‐report questionnaire data and Game Craving Scale scores. In the probability‐selection task, behavioral data from the learning and test trials entered a repeated‐measures analysis of variance (ANOVA) with the within‐subjects factors Block (1–3), Pair (AB, CD, EF), and the between‐subjects factor Group (IGD vs. HC); and behavioral data from the transfer trials without feedback entered a repeated‐measures ANOVA with the within‐subjects factors Conditions (high reward pairing; High loss pair) and the between‐subjects factor Group (IGD vs. HC). The dependent variables were the reaction time and accuracy. In the Stop signal task, behavioral data entered a repeated‐measures ANOVA with the within‐subjects factors Clue Type (Reward; Loss; Neutral) and the between‐subjects factor Group (IGD vs. HC). And the dependent variables were the reaction time and accuracy of the Go trial, SSD and SSRT of Stop trial. Stop Signal Reaction time (SSRT) is considered a key indicator of measured behavioral inhibition ability in stop signal task. SSRT was calculated by subtracting the critical SSD from the RT in the Go trials. The longer SSRT indicates a poor reaction inhibition. According to the tracking algorithm, Stop signal delay (SSD) is the time interval between the Go signal and the STOP signal (Williams et al., 1999).

## RESULTS

Table 1 shows the demographic variable and questionnaire data of the IGD and HC groups. Through data analysis, it can be seen that there is no significant difference in age between the two groups, but there are significant differences in scores of DSM‐5, IAT, weekly game duration and game craving (*p < *.001).

### Reward learning result

**FIGURE 2:**
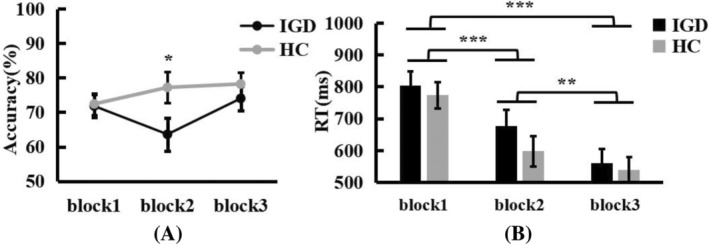
(A) Line chart of the accuracy of reinforcement learning phase for three blocks. (B) Line chart of the reaction time (RT) of reinforcement learning phase for three blocks. Asterisks represent significant differences according to post hoc Bonferroni tests at the **p < .01, ***p < .001 levels. HC, healthy control group; IGD, Internet gaming disorder group.

A mixed‐design ANOVA of the correct rate (the correct rate is the probability of the participants choosing A, C, or E stimulation) with factors Group (IGD vs. HC) and Pair (AB vs. CD vs. EF) and Block (block1 vs. block2 vs. block3). There is no significant effect of Group, *F*(1,41) = 1.958, *p = *.69, *η* ^*2*^ = .046. The main effect of Pair was significant, *F*(2,82) = 26.105, *p < *.001, *η* ^*2*^ = .389, with the correct rate of AB higher than CD (*p = *.039) and EF (*p < *.001), and that of CD higher than EF (*p < *.001). The main effect of Block is not significant, *F*(2,82) = 2.324, *p = *.04, *η* ^*2*^ = .054. Furthermore, the interaction between Group and Pair was not significant, *F*(2,82) = 0.628, *p = *.536, *η* ^*2*^ = .015. The interaction between Group and Block was significant, *F*(2,82) = 3.003, *p = *.055, *η* ^*2*^ = .068. A simple effects analysis revealed that in the second block, the correct rate of IGD participants was significantly lower than that of HC participants (*p = *.047), and the IGD participants correct rate of the third block was higher than that of the second block (*p = *.059). The interaction between Pair and Block was significant, *F*(4,164) = 2.426, *p = *.050, *η* ^*2*^ = .056. A simple effects analysis revealed that the correct rate of AB pair was significantly higher than that of EF pair in block1 (*p < *.001), AB pair (*p < *.001; *p < *.001), and CD pair (*p = *.001; *p < *.001) in block2 and block3 was higher than EF, AB in the third block was higher than the second block (*p = *.053), and CD in the third block was higher than the first block (*p = *.004) and the second block (*p = *.030). There was no significant interaction among them, *F*(4,164) = 1.197, *p = *.314, *η* ^*2*^ = .028 (Figure 2A).

A mixed‐design ANOVA of the reaction time with factors Group (IGD vs. HC) and Pair (AB vs. CD vs. EF) and Block (block1 vs. block2 vs. block3) showed that there was no significant difference between groups, *F*(1,41) = 0.576, *p = *.452, *η* ^*2*^ = .014. The main Pair effect was significant, *F*(2,82) = 11.343, *p < *.001, *η* ^*2*^ = .217. Post hoc analysis showed that the reaction times of CD pair (*p = *.024) and EF pair (*p < *.001) were higher than that of AB pair. The main effect of Block was significant, *F*(2,82) = 49.448, *p < *.001, *η* ^*2*^ = .547, with the reaction time of block1 higher than that of block2 (*p < *.001) and block3 (*p < *.001), and that of block2 higher than that of block 3 (*p = *.002). There were no significant interactions between Group and Pair, *F*(2,82) = 0.531, *p = *.590, *η* ^*2*^ = .013. There was no significant interaction with Group and Block, *F*(2,82) = 0.813, *p = *.447, *η* ^*2*^ = .019 (Figure 2B). And there was no significant interaction with Block and Pair, *F*(4,164) = 0.172, *p = *.953, *η* ^*2*^ = .004. There was no significant interaction among them, *F*(4,164) = 1.582, *p = *.82, *η* ^*2*^ = .037.

### Reward test stage

**FIGURE 3:**
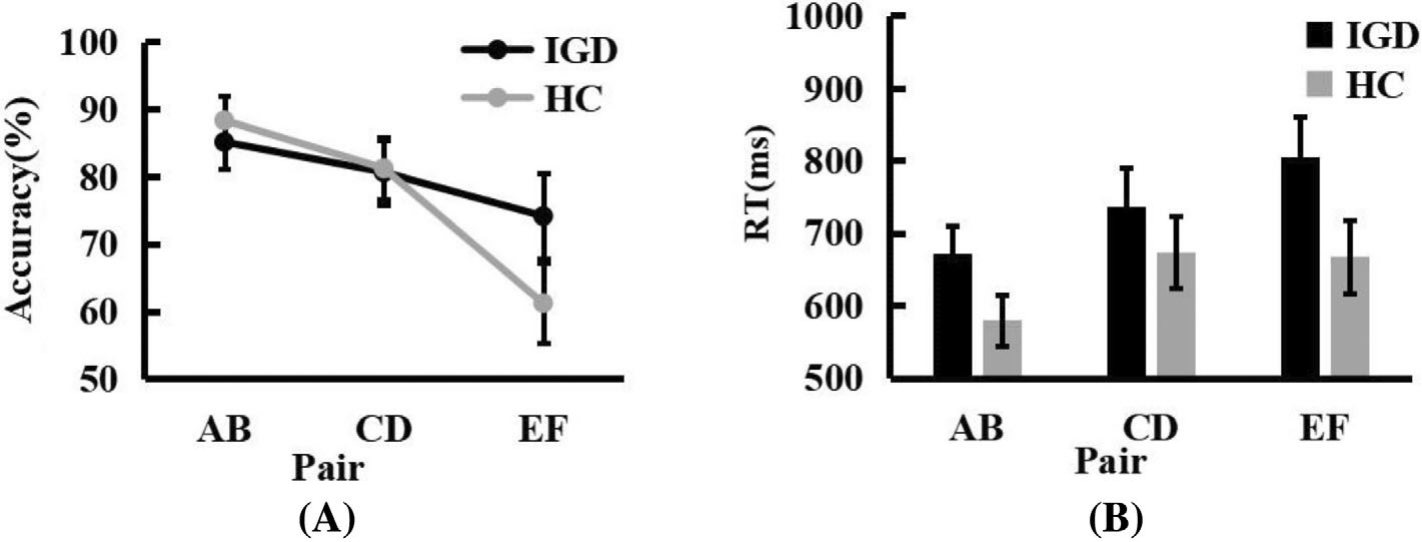
(A) Line chart of the accuracy of test phase for three pairs. (B) Line chart of the reaction time (RT) of test phase for three pairs. Asterisks represent significant differences according to post hoc Bonferroni tests at the *p < .05, **p < .01, ***p < .001 levels. HC, healthy control group; IGD, Internet gaming disorder group.

A mixed‐design ANOVA of the correct rate with factors Group (IGD vs. HC) and Pair (AB vs. CD vs. EF) showed that there was no significant difference between Group, *F*(1,41) = 0.385, *p = *.538, *η* ^*2*^ = .009. Pair effect was significant, *F*(2,82) = 10.315, *p < *.001, *η* ^*2*^ = .201. Post hoc analysis showed that the correct rate of EF pair was significantly lower than that of AB pair (*p < *.001)and CD pair (*p = *.046). The interaction between them was not significant, *F*(2,82) = 2.018, *p = *.40, *η* ^*2*^ = .047 (Figure 3A).

A mixed‐design ANOVA of the reaction time with factors Group (IGD vs. HC) and Pair (AB vs. CD vs. EF) showed that the effect between Group was not significant, *F*(1,41) = 2.499, *p = *.22, *η* ^*2*^ = .057. The main Pair effect was significant, *F*(2,82) = 12.127, *p < *.001, *η* ^*2*^ = .228, with the CD pair (*p = *.006) and EF pair (*p < *.001) higher than the AB pair. There were no significant interactions between them, *F*(2,82) = 1.334, *p = *.269, *η* ^*2*^ = .032 (Figure 3B).

### Reward learning transfer stage

**FIGURE 4:**
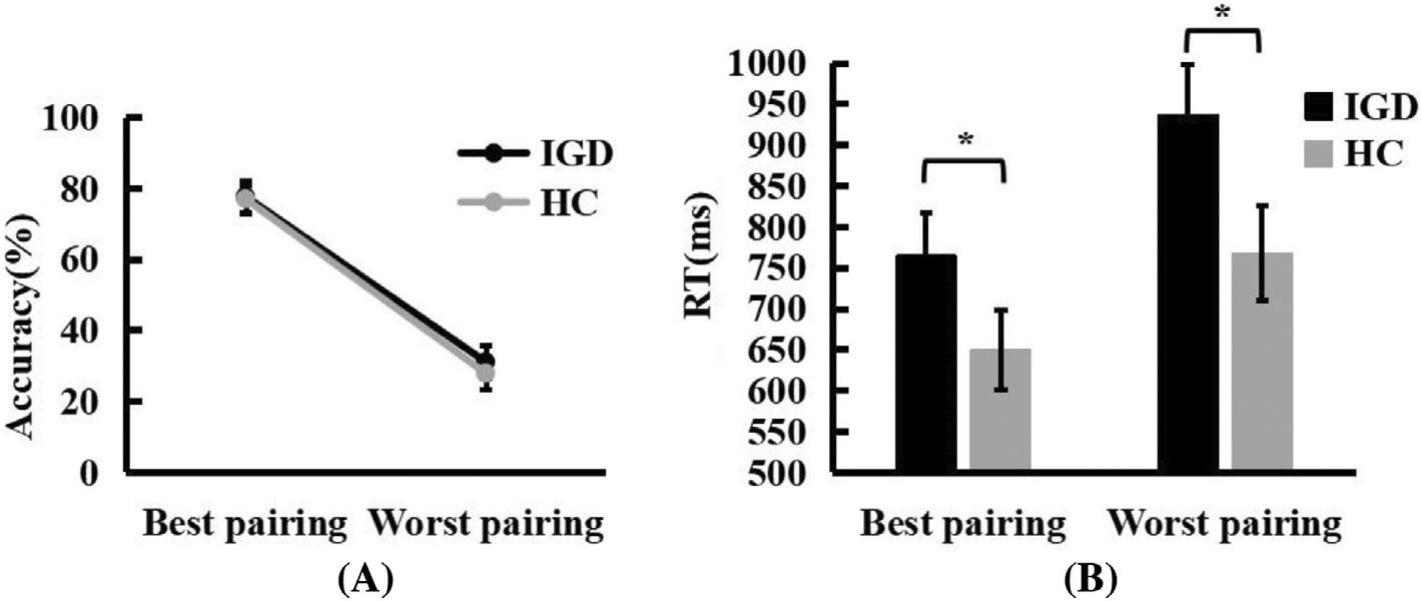
(A) Line chart of the accuracy of transfer phase. (B) Line chart of the reaction time (RT) of transfer phase. The asterisk indicates that the reaction time of the addicted group is slightly higher than that of the HC group (*p* = .064). Asterisks represent significant differences according to post hoc Bonferroni tests at the *p < .05, **p < .01, ***p < .001 levels. HC, healthy control group; IGD, Internet gaming disorder group.

A mixed‐design ANOVA of the correct rate with factors Group (IGD vs. HC) and Condition (High‐Reward Pair vs. High‐Loss Pair) showed that there was no significant difference between Group, *F*(1,41) = 0.259, *p = *.613, *η* ^*2*^ = .006. The main effect Condition was significant, *F*(1,) = 90.638, *p < *.001, *η* ^*2*^ = .689, and the correct rate of high‐reward pair was significantly higher than that of high‐loss pair (*p < *.001). The interaction between them was not significant, *F*(1,41) = 0.063, *p = *.803, *η* ^*2*^ = .002 (Figure 4A).

A mixed‐design ANOVA of the reaction time with factors Group (IGD vs. HC) and Condition (High‐Reward Pair vs. High‐Loss Pair) showed that the Group was significant, *F*(1,41) = 3.624, *p = *.064, *η* ^*2*^ = .081. The reaction time of IGD participants was higher than HC participants (*p = *.056). The main effect Condition was significant, *F*(1,41) = 36.107, *p < *.001, *η* ^*2*^ = .468, and the reaction time of high‐loss pairing was significantly higher than that of high‐reward pairing (*p < *.001). There was no significant interaction between them, *F*(1,41) = 1.280, *p = *.264, *η* ^*2*^ = .030 (Figure 4B).

### Stop signal task result

In the stop signal task, for the accuracy of the Go trial, we conducted a 2 (Group: IGD vs. HC) × 3 (Clue: Reward vs. Loss vs. Neutral) mixed‐design ANOVA. The results show that the main effect of Clue was significant, *F*(2,82) = 4.145, *p = *.021, *η* ^*2*^ = 0.090. The reward clue was higher than the loss clue (*p = *.040) and neutral clue (*p = *.047). There was no significant difference between Group, *F*(1,41) = 0.898, *p = *.444, *η* ^*2*^ = .014. There was no significant interaction between them, *F*(2,82) = 0.667, *p = *.516, *η* ^*2*^ = .016.

In the stop signal task, for the reaction time of the Go trial, we conducted a 2 (Group: IGD vs. HC) × 3 (Clue: Reward vs. Loss vs. Neutral) mixed‐design ANOVA. The results show that the main effect of Clue was significant, *F*(2,82) = 4.567, *p = *.013, *η* ^*2*^ = .100, and the reaction time of loss clue was higher than that of neutral clue (*p = *.078). There was no significant difference between Group, *F*(1,41) = 0.092, *p = *.764, *η* ^*2*^ = .002. And there was no significant interaction between them, *F*(2,82) = 2.320, *p = *.05, *η* ^*2*^ = .054.

**FIGURE 5:**
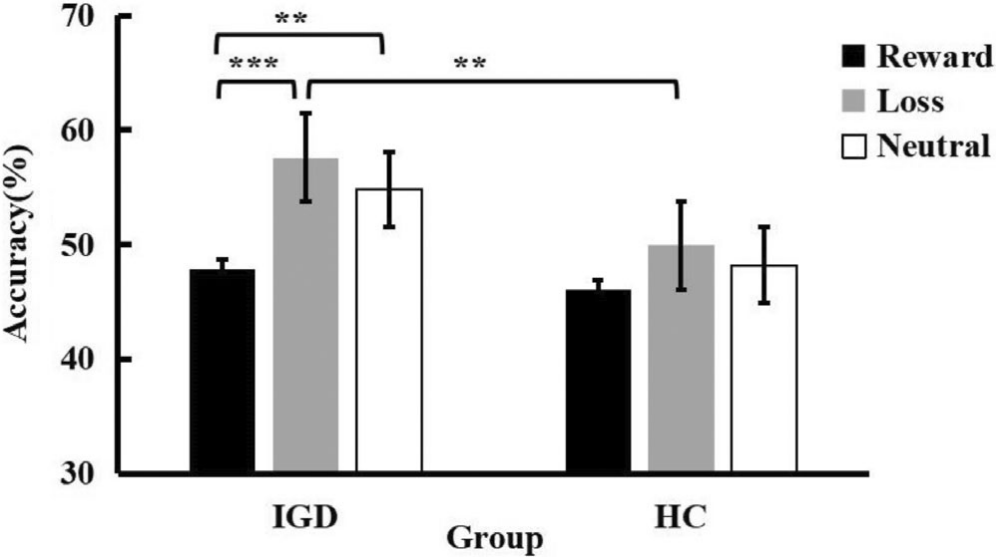
The correct rate of signal stop task in No‐Go trials. Asterisks represent significant differences according to post hoc Bonferroni tests at the **p < .01, ***p < .001 levels. HC, healthy control group; IGD, Internet gaming disorder group.

For the accuracy of the no‐go trial, we conducted a 2 (Group: IGD vs. HC) × 3 (Clue: Reward vs. Loss vs. Neutral) mixed‐design ANOVA. The results showed that the main effect of Clue was significant, *F*(2,82) = 9.049, *p < *.001, *η* ^*2*^ = .181. The loss clue (*p = *.001) and neutral clue (*p = *.009) were significantly higher than the reward clue. There was no significant difference between Group, *F*(1,41) = 3.488, *p = *.069, *η* ^*2*^ = .078. The interaction between them was significant, *F*(2,82) = 3.080, *p = *.051, *η* ^*2*^ = .070. After simple effect test, it was found that the correct rate of reward clue was lower than that of loss clue (*p < *.001) and neutral clue (*p = *.003) in IGD participants, but there was no significant difference among the three conditions in the HC participants. And only under the clue of loss, the correct rate of IGD participants (*p = *.005) was higher than that of the HC participants (Figure 5).

The result of SSD analysis shows that the main effect of Clue was not significant, *F*(2,82) = 1.595, *p = *.209, *η* ^*2*^ = .037. There was no significant difference between group, *F*(1,41) = 1.199, *p = *.280, *η* ^*2*^ = .028. And there was no significant interaction between them, *F*(2,82) = 0.374, *p = *.689, *η* ^*2*^ = .009.

The results of SSRT analysis show that the Clue main effect was significant, *F*(2,82) = 6.261, *p = *.003, *η* ^*2*^ = .132. The reward clue (*p = *.069) and loss clue (*p = *.033) were higher than that of neutral clue. There was no significant difference between Group, *F*(1,41) = 1.778, *p = *.90, *η* ^*2*^ = .042, and there was no significant interaction between them, *F*(2,82) = 0.176, *p = *.839, *η* ^*2*^ = .004.

## DISCUSSION

By discussing the influence of reward learning on subsequent inhibition control among IGD individuals, the research had the following findings.

### Performance of reinforcement learning for IGD individuals

This study evaluated the ability of reinforcement learning of the IGD group by probabilistic selection task. It was observed that the frequency with which two groups chose reward‐associated stimuli exceeded 50% significantly in two stages of reinforcement learning and learning test. These results indicated that all participants had acquired this learning task, approaching to reward‐related stimuli and avoiding punishment‐related stimuli. Two groups had established successfully the same level of connection with different probability stimuli.

However, the IGD group needed to spend more trials establishing the connection between neutral and reward/punishment stimuli, reaching the same level as that of the HC group. In the second block of reinforcement learning phase, the accuracy of the IGD group was lower than that of the HC group, but there were no significant differences in the first block, the third block or the learning test. This result was consistent with our previous hypothesis that the ability of reinforcement learning of IGD individuals was impaired. Previous investigations also found similar results in participants with other types of addiction disorder. For example, substance abusers with alcohol, marijuana or nicotine performed worse in associative learning with reward and punishment (Addicott et al., 2019; Koob, 2013; Koob & Vendruscolo, 2023; Zhou et al., 2023). The decrease of dopaminergic (DA) activity in the limbic of the midbrain was the core feature of addiction, which might be one of the important factors of producing IGD. DA in the limbic system was involved in hedonic experience (Ikemoto & Panksepp, 1999), reward prediction (Schultz et al., 1997), and reinforcement learning based on reward prediction errors (Myers et al., 2016). Individuals with IGD showed insensitivity to rewards and losses, which could lead them to adjust difficultly according to negative feedback, and induce excessive game behaviors. Therefore, the impaired ability of learning and adaption from feedback might be used to explain the poor academic performance of teenagers and young people with IGD (Jeong & Kim, 2011).

### Adaptation of reward/punishment‐related stimuli in the new environment for IGD individuals

Participants with IGD were slower in adapting to the new contexts with reward/punishment‐associated stimuli. In transitive inference test, the reaction time to reward/punishment‐associative stimuli pairs in the IGD group was longer than that of the HC group. This slower adaptation was not caused by the different levels of reinforcement learning, because there were no significant differences between two groups in the phase of the learning test. In the new reward/punishment‐related stimuli pairs, individuals with IGD needed more time to identify and select stimuli, which indicated the decreased flexibility of game addition. Previous investigations also found similar results in participants with other types of addiction disorder. For example, substance abusers with alcohol, marijuana or nicotine performed worse in reversal learning with reward and punishment (Bağci et al., 2022; Leppink et al., 2016; Nesic et al., 2011). This result in reward transfer test is also consistent with many research findings. Cognitive flexibility in online game addicts was impaired (Zhou et al., 2012). IGD individuals play games repeatedly or even uncontrollably, and they fail to flexibly switch from game situations to daily life. In addition, addiction can be defined as a maladaptive decision‐making process. In this process, addicts persistently seek some reward stimuli in spite of the potential loss risk or other negative consequences. Updating emergency measures for rewards and losses is a basic aspect of effective decision‐making, and they need to adapt to the changing environment (Hong et al., 2023; Zhang et al., 2023).

### Inhibitory control behavior of IGD to neutral stimuli

There were no significant differences between the two groups in the neutral signal stop task, which suggested that the inhibitory control ability to neutral stimuli of two groups had no differences.

However, this behavioral result might not mean that the inhibitory ability to neutral stimuli of the IGD group was the same as that of the HC group. Investigations found that there were obvious impairments of cognitive function and inhibitory control for individuals with IGD. In the color–word Stroop task, adolescents with IGD made more mistakes than the HC group under inconsistent conditions, which indicated that the inhibitory control of IGD participants might be worse (Cai et al., 2016; Xing et al., 2014; Yuan et al., 2016). In another inhibitory task of Go/No‐Go task, patients with IGD performed worse (Kräplin et al., 2020; Yan et al., 2021). Some studies also found that there was no significant behavioral difference in the Go/No‐Go task between the IGD group and the HC group, but there was a difference in the activation of brain regions. Compared with the HC group, the inhibition circuit of the frontal striatum in the IGD participants was defective (Chen et al., 2015; Ko et al., 2014). A study using functional magnetic resonance imaging (fMRI) has also confirmed this conclusion, and found that the inhibition control performance was worse in the Go/No‐Go task and impaired prefrontal function might relate to high impulsivity in adolescents with IGD, which might contribute directly to the Internet addiction process (Chen et al., 2021; Ding et al., 2014).

### Effect of reinforcement learning on inhibitory control response of IGD

Previous reward‐related learning improved the approaching behavior response to reward‐related stimulation, and there was no difference between the IGD group and the HC group. Specifically, the accuracy of the Go signal shows that the reward cue is higher than the other two cues, which indicates that the reward can promote both groups. One explanation is that when encountering the previous reward prediction stimulus, the approach tendency will be generated automatically because the response to these rewards is favorable (Anderson et al., 2016). A previous study showed that the neural activity in the motor control area of the prefrontal cortex was increased by unrelated reward prediction stimulation, that is, when an individual was stimulated by reward, this reaction tendency would be triggered automatically (Krebs et al., 2011).

The selectivity of reward learning weakens the inhibitory behavior response of the IGD group to reward‐related stimulation, and improves the avoidance behavior response of the IGD group to loss‐related stimulation. Specifically, the correct rate of no‐go is lower in the IGD group under the reward cue than in the loss cue and neutral cue, which indicates that the IGD group has difficulty suppressing the response to previous rewards. This shows that previous reward learning has more influence on IGD individuals' ability to inhibit reward‐related stimuli, which not only makes it easier to capture reward‐related stimuli, but also makes it more difficult to inhibit reward‐related stimuli. One reason may be the imbalance between goal‐oriented system and habit‐oriented system of IGD patients (Zhou et al., 2021). Previous studies have shown that, in the habit test of tool learning paradigm, Internet addicts will persist in responding to the previous reward stimulus even if the reward results do not appear (Zhou et al., 2018). This persistence shows that IGD groups are more inclined to develop reward‐driven habits, and tend to rely too much on these habits, so they fail to adjust their behavior in the process of target revaluation. Moreover, in previous studies on alcohol addiction, compared with the HC group, the sensitivity of alcohol‐dependent patients to reward devaluation was significantly reduced (Sjoerds et al., 2013). That is to say, they rely too much on inflexible habit systems. Most existing studies show that the disruption of this balance leads to the maintenance of addiction (Belin et al., 2013; Everitt & Robbins, 2016).

The IGD group suppressed loss‐related stimuli better, which may indicate that the IGD group is insensitive to loss cues. This study found that the correct rate of the IGD group under loss clues was higher than that of HC group, which indicated that the IGD group had no impulse to start when faced with loss. A mixed gambling task study showed that the IGD group's loss sensitivity decreased (Raiha et al., 2020). In addition, data from fMRI demonstrated that male players showed more sensitivity to rewards and less sensitivity to losses while they were performing a card‐guessing task (Zhang et al., 2020). An event‐related potential (ERP) investigation found that the FRN and P300 amplitudes of negative feedback produced for IGD participants decreased (Balconi et al., 2017). At the same time, the activation of the anterior cingulate cortex of IGD participants to negative feedback decreased, which indicated that the sensitivity of IGD population to loss decreased (Pan et al., 2018; Zhang et al., 2020). The IGD group is insensitive to loss, which leads to the inability to realize that negative stimulation starts slowly and reacts slowly to negative consequences, resulting in excessive game play.

However, previous intensive learning had no significant effect on the inhibitory behavior of the HC group. This shows that the previous reward learning only destroyed the inhibition and control ability of the IGD group; however, for healthy people, when the goal orientation conflicts, they can restrain themselves well and react correctly. Facts have proved that human beings can flexibly adjust their behavior in relatively simple laboratory tasks. For example, a series of recent experiments show that after over training stimulus–response‐result correlation, when the result changes accidentally, human beings continue to choose the right action (Luque et al., 2020). Another study shows that participants can overcome the influence of Pavlov's learning in a simple inhibition task, and it is believed that the possible situational characteristics and individual differences determine whether the reward history will significantly affect the process of behavior activation and inhibition, thus leading to non‐beneficial behaviors (Marchner & Preuschhof, 2022).

Abnormal reward learning function may be an important reason for the formation and maintenance of IGD. During the game process, game players' game‐related stimuli are gradually given different reward values, and individuals can predict the upcoming game experience after establishing stimulus–response connection (Kim et al., 2021). These game rewards and game‐related clues make the reward system a highly active state. In the visual search task, IGD individuals showed sustained attention to game‐related pictures compared with neutral stimuli (Heuer et al., 2021). Especially after a period of abstinence, IGD individuals show a stronger automatic retrieval bias towards game‐related stimuli (Zheng et al., 2022). Addicts always automatically capture the game reward information in the environment and are constantly attracted to it, which make it difficult for IGD individuals to quit the game. Therefore, the IGD group's difficulty in suppressing the search for previous rewards and insensitivity to loss may be the important reasons for its addiction maintenance.

### Limitations

This study is the first to combine the reward and inhibition control systems to explore the impact of reward learning performance on subsequent inhibition control in an IGD group, which illustrates the link between the two systems. Compared with the HC group, the accuracy of the IGD group was significantly lower in the reward learning phase. At the same time, the IGD group was slower in adapting to the new contexts and previous reward learning performance might have caused subsequent inhibition control deficiency.

However, there are some limitations in this study. First of all, this study only explored the behavioral influence of reward learning on the IGD group's inhibition control, but there was no in‐depth study on the neural basis of the former's influence on inhibition control. The EEG study of loss‐related stimulation in reward learning stage and subsequent inhibition control can better reveal whether IGD individuals passivate loss‐related stimulation in these two stages. Second, this study only found that the reward learning ability of IGD was impaired from a cross‐sectional perspective, but did not explore whether the reward learning ability was the cause of IGD from a vertical perspective. At present, some studies have found that rewarding learning ability can predict addictive behavior (Rai et al., 2019; Villiamma et al., 2022). The exploration of the neurobiological mechanism behind the change of reward learning can provide new insights into the biological behavior mechanism that mediates addiction susceptibility. Finally, the subjects used in this study were college students, and further research should verify whether it is suitable for teenagers or other age groups. Teenagers are more continuously disturbed by previous reward relationships than adults (Roper et al., 2014). This may lead to a more lasting destructive effect of reward on teenagers than adults, and teenagers are in a high incidence of various risky behaviors, so more attention should be paid to teenagers' online game addiction in the future.

## CONFLICT OF INTEREST STATEMENT

The authors have declared that they have no conflicts of interest.

## ETHICS STATEMENT

We addressed ethical concerns by the following: (1) Informed consent was obtained from all participants involved in the study. (2) The current research was done according to the ethical standards set out in the Declaration of Helsinki in 1964 and its later amendments, or comparable ethical standards. (3) The research protocol was approved by the Psychology Research and Ethics Committee at Henan University in China (project identification code: 202109306).

## Supporting information


**Data S1:** Supporting Information.
