# Supplementary material for: Influence of reinforcement learning on the inhibitory control of Internet gaming disorder
Source: Psych J. 2024 Jul 5;13(6):966–78. doi: 10.1002/pchj.772 (PMC11608794; doi:10.1002/pchj.772)
Supplement: Supplementary file 1 — Data S1: Supporting Information. [file PCHJ-13-966-s001.pdf]

Thank you very much for your email with which you sent us the reviewer's report on Manuscript ID PsyCh-2023-251. We also wish to take this opportunity to thank the reviewer for his valuable comments and have revised the manuscript carefully.

Question1:

Please check the document carefully for editing. Please ensure that there is a space between the word and the citation. There are some instances in the document where this is not the case. There are some other spacing issues too.

Relay: Thanks for your suggestion, and errors have been amended in the new manuscript.

Question 2:

When referring to the DSM-V, please consider using DSM-V-TR? Or indicate why not?

Relay: We are very grateful for your comments on the manuscript. The DSM-V-TR is the latest version of Diagnostic and Statistical Manual of Mental Disorders. In addition, the nine diagnostic criteria of DSM-V-TR are the same as the nine diagnostic criteria of DSM-V. Therefore, we decided to adopt the DSM-V-TR in the new text and the relevant parts were revised.

"Internet Gaming Disorder was included in the Text Revision of Diagnostic and Statistical Manual of Mental Disorders Fifth Edition (DSM-V-TR), which marked Internet Gaming Disorder officially as a psychological disorder."

"All participants were selected based on a modified Internet addiction test (IAT) (Young, 1996) and the nine-item diagnostic criteria in the DSM-V-TR (American Psychological Association, 2013). IGD participants scored higher than 50 on the

modified IAT and concurrently met five or more DSM-V-TR criteria, and played Internet games for minimum 14 hr per week during the last 2 years. Health control (HC) scored lower than 50 on modified IAT and met less than five DSM-5V-TR criteria, and played online games for less than 1 hr per day. ”

“The Diagnostic and Statistical Manual of Mental Disorders-5 (DSM-5) is based on the nine diagnostic criteria of DSM-V (Petry et al., 2014). Each item corresponds to a symptom of online game addiction, and a score of more than 5 meets the DSM-V online game addiction screening standard. Specially, the nine diagnostic criteria of DSM-V-TR are the same as the nine diagnostic criteria of DSM-V.”

Question 3:

I strongly recommend language editing for the whole document.

Relay: Thanks for your advice on the text, we have amended the relevant parts in the new manuscript.
